# Development of Soft Luliconazole Invasomes Gel for Effective Transdermal Delivery: Optimization to In-Vivo Antifungal Activity

**DOI:** 10.3390/gels9080626

**Published:** 2023-08-03

**Authors:** Sunitha Kumari, Omar Awad Alsaidan, Dibyalochan Mohanty, Ameeduzzafar Zafar, Swagatika Das, Jeetendra Kumar Gupta, Mohammad Khalid

**Affiliations:** 1 Department of Pharmaceutics, Anurag University, Hyderabad 500088, Telangana, India; sunithayadav.sy@gmail.com; 2 Department of Pharmaceutics, College of Pharmacy, Jouf University, Sakaka 72341, Al-Jouf, Saudi Arabia; osaidan@ju.edu.sa (O.A.A.); azafar@ju.edu.sa (A.Z.); 3 School of Pharmacy, Centurion University of Technology and Management, Gopalpur 756044, Odisha, India; swagatikadas.med@gmail.com; 4 Institute of Pharmaceutical Research, GLA University, Mathura 281406, Uttar Pradesh, India; jk.gupta@gla.ac.in; 5 Department of Pharmacognosy, College of Pharmacy, Prince Sattam Bin Abdulaziz University, Al-Kharj 11942, Riyadh, Saudi Arabia

**Keywords:** transdermal delivery, luliconazole, invasomes gel, ex vivo skin permeation, antifungal activity

## Abstract

Luliconazole (LZ) is a good candidate for the treatment of fungal infection topically but has limitations, i.e., poor solubility and poor permeability to skin. Due to these limitations, multiple administrations for a long time are required to treat the inflection. The aim of the present study was to develop the invasomes (IVS) gel of LZ to improve the topical antifungal activity. The IVS was prepared by the thin-film hydration method and optimized by Box-Bhekhen design software. The optimized LZIVS (LZIVSopt) has 139.1 ± 4.32 nm of vesicle size, 88.21 ± 0.82% of entrapment efficiency, 0.301 ± 0.012 of PDI, and 19.5 mV (negative) of zeta potential. Scanning microscopy showed a spherical shape of the vesicle. FTIR spectra showed there is no interaction between the drug and lipid. Thermogram showed that the LZ is encapsulated into the LZIVS matrix. LZIVSopt gel (LZIVSopt-G3) exhibited optimum viscosity (6493 ± 27 cps) and significant spreadability (7.2 g·cm/s). LZIVSopt-G3 showed 2.47-fold higher permeation than pure LZ-gel. LZIVSopt-G3 did not show any edema or swelling in the skin, revealing that the developed formulation is non-irritant. LZIVSopt-G3 exhibited significant inhibition of the fungus infection (*C. albicans*) in the infected rats. The finding concluded that IVS gel is a good carrier and an attractive approach for the enhancement of topical delivery of LZ to treat the fungal infection.

## 1. Introduction

Fungal infections are a serious health issue and a significant contributor to morbidity (1.7 million/year worldwide, Kainz et al., 2020 [1]. There are two types of fungal infections: superficial and invasive. Up to 20–25% of the world’s population is susceptible to superficial fungal infections, which can interfere with everyday life and lead to higher healthcare costs [2]. Dermatophytes, Candida, and Malassezia species infections are all known fungi, which are the causes of superficial fungal infections [3,4]. The antifungal agents that are now on the market block the formation of ergosterol (the most plentiful sterol in fungal cell membranes) into fungi and inhibited the growth or multiplication of cell membranes [5]. Luliconazole (LZ) is a topical antifungal drug and chemically belongs to an imidazole category. LZ inhibited the cell membrane synthesis of the fungus and was more efficacious than the other present imidazole [6]. LZ was approved by the United States food drug administration (USFDA) in 2013 and is available in the market in the form of cream (1% LZ) [3]. LZ is a poor water-soluble drug (0.065 mg/mL at 25 °C) and belongs to the biopharmaceutical classification system-II (BCS-II) [7,8]. LZ is used for the treatment of various fungal infections, such as Tinea pedis, Tinea cruris, and Tinea corporis, which are caused by the fungi T. rubrum and E. floccosum [9]. However, the commercial topical preparation of LZ (1% *w*/*v* cream LUZU^®^) is not significantly properly permeated or absorbed through the skin due to low residence time or less crossing of the stratum corneum layer of the skin [10]. Thus, it has been required to develop a novel drug delivery system (nanotechnology-based) for the improvement of the therapeutic activity of drugs. There are various nanotechnology gel-based formulations for transdermal delivery have been reported by researchers to improve the therapeutic activity of the drugs such as fusidic acid-loaded nanoemulgel [11], Fluconazole solid lipid nanoparticles gel [12], griseofulvin-loaded solid lipid nanoparticles laden gel [13], amphotericin B-loaded Nanostructured Lipid Carriers gel [14], fluconazole loaded liposomes gel [15], tolnaftate pro-vesicular gel [16], clotrimazole loaded ethosomal gel [17], and Agomelatine-loaded IVS gel [18]. The vesicular delivery systems (liposomes, niosomes, ethosomes, invasomes, etc.) are the potential drug carrier for topical delivery because of their distinct benefits (high permeation) and excellent flexibility [19]. Among them, invasomes (IVS) are the novel vesicular carrier of drugs for transdermal delivery and enhanced penetration than conventional liposomes [20]. IVS is flexible vesicles and composed of phospholipids, alcohol, and terpenes. Alcohol has the property that enhances the flexibility of lipids bi-layer of IVS, producing soft vesicles leading to increasing skin permeability of the drug [21]. Ethanol can fluidize and disturb the stratum corneum lipid leading to an increase in the permeability of the drug [22]. Terpenes also enhance the skin permeation of both lipophilic and hydrophilic drugs by re-arrangement of the stratum corneum layer of skin [23,24]. Incorporation of IVS into the gel system provided the sustained release of the drug and increased the contact time (residence time) of formulation over the skin as well as penetration [25,26,27]. There are many reports published on IVS gel, which improved the transdermal delivery of drugs. Hoda and its associates developed an itraconazole-loaded IVS gel for the treatment of topical fungal infection. Formulation exhibited significantly higher ex vivo permeation and better antifungal activity than marketed preparation [28]. Gupta’s group formulated the ketoconazole invasomes gel for the treatment of fungal infection and showed high skin permeation as well as no irritation tested on rabbit skin [29]. Carbopol 934 is a water-soluble polymer used as a gelling agent for the delivery of therapeutic agents through topical application. It is a white powder and crosslink polyacrylic acid polymer. It is a non-toxic, non-irritating, and biodegradable polymer [30]. The present research work developed the LZ-loaded IVS and incorporated it into Carbopol 934 gel. The novelty of the present research work is to increase skin permeability by re-arrangement of the stratum corneum layer of skin and therapeutic efficacy of LZ in LZ-IVS gel formulation. The IVS was prepared by the thin-film hydration method and optimized by Box-Bhekhen design software. Box-Bhekhen design is a commonly used method for the optimization of pharmaceutical formulations. It is a second-order response surface methodology. The three levels of the independent variable (low, middle, and high) require observed influence on the responses. It provided a smaller number of experiments run in different compositions of formulation factors as well as prevented the wastage of time and chemicals [31]. The LZ-IVS formulation was evaluated for vesicle size (VS), entrapment efficiency (EE, %), and scanning electron microscopy. Further, the optimized LZ-IVS was incorporated in different concentrations of carbopol gel and evaluated for viscosity, spreadability, in vitro release, ex vivo skin permeation, toxicity, and antifungal activity.

## 2. Materials and Methods

### 2.1. Materials

Luliconazole was purchased from Manus Aktteva Biopharma (Ahmedabad, Gujarat, India). Soy lecithin, citronella oil, ethanol, and Carbopol 934 were purchased from SD Fine Chem (Mumbai, India). A dialysis bag (MWCO 12 kDa) was purchased from the HiMedia Laboratories (Mumbai India). All other chemicals used for the study and obtained from the laboratory are analytical grads.

### 2.2. Optimization of Nano Invasomes

The LZ-IVS formulations were optimized by using experimental design software [31] after preliminary examination. The 3-factor and 3-level box Box-Bhekhen design (Design–Expert 9.0.3.1 software, Stat-Ease Inc., Minneapolis, MN, USA) was applied. The phospholipid (X_1_), terpene (Citronella oil, X_2_), and sonication time (X_3_) were selected as independent variables, while VS, (Y_1_), EE, (Y_2_), and polydispersity index (Y_3_) were taken as dependent variables. A total of thirteen experimental runs (Table 1) were obtained from the software. The LZ-IVS formulations were prepared, and responses of each LZ-IVS were applied to different experimental models (linear, 2F, and quadratic) to determine the best-fitted model and analysis the statistical regression analysis. The polynomial equation, 3-dimensional, and contour plot were constructed to examine the impact of formulation variables on the responses (PS, PDI, and EE). Further, the optimized LZ-IVS formulation was selected from the point prediction of the software by varying the composition of ingredients from the center point composition of IVS and desirability function.

### 2.3. Preparation of Luliconazole-Loaded Invasomes

LZ-loaded IVS formulations were developed by the thin-layer hydration method [32]. The required quantity of phospholipid and Citronella oil were weighed (Table 1) and transferred into the round-bottom flask. Then, it was dissolved using methanol and chloroform (2:1 *v*/*v*). The LZ (50 mg) was added to the lipid solution. Then, the flask was fixed into a rotary evaporator (KIA, RV3, Staufen, Germany), and the organic solvent was removed at 45 °C under reduced pressure. The thin film was formed and placed in a vacuum desiccator overnight to remove the moisture. Then formed lipid film was hydrated with ethanolic–phosphate buffer saline (pH 7.4) in a rotary evaporator (60 rpm) at 40 °C for 1 h. Then, the developed IVS was stood for 2 h at 25 °C to get multilamellar vesicles. Further, IVS was sonicated using a probe sonicator (Sonics Vibra cell, Church Hill Rd, Finleyville, PA 15332, USA) at 4 °C at 40% output frequency (40 W) for 3–5 min at 30-s intervals and then stored at 4 °C until further evaluation.

### 2.4. Characterization of Vesicle

#### 2.4.1. Vesicles Size and Polydispersity Index and Zeta Potential Analysis of LZIVS

The VS, PDI, and zeta potential of the LZIVS were measured using the dynamic light-scattering method (Malvern Zetasizer Malvern, UK) at 25 °C and 90° scattering angle using the water as a reference medium (RI = 1.33). The LZIVS formulation was diluted 20 times with distilled water and filled into the cuvette. The cuvette was placed into Zetasizer, and the VS, PDI, and zeta potential were measured [28].

#### 2.4.2. Entrapment Efficiency

The EE of LZ in LZIVS was determined by the ultra-centrifugation method. LZIVS formulations were filled in a centrifugation tube and centrifuged at 15,000 rpm for 4 °C (REMI-24, cooling centrifuge, Mumbai, India). The supernatant was separated, and absorbance was analyzed by UV-spectrophotometer (UV-3200, LabIndia Analytical, Thane West, Maharashtra, India) at 297 nm after appropriate dilution. The % *EE* was calculated by the given equation [33].

(1) $$\%\;EE=\frac{Ct-Cf}{Ct}\times 100$$

where *Ct* = total concentration of luliconazole and *Cf* = concentration of free luliconazole.

#### 2.4.3. Scanning Electron Microscopy (SEM)

The morphology of optimized LZIVS (LZIVSopt) was analyzed by SEM (SEM-HITACHI, Tokyo, Japan) at 20 kV energy. The LZIVSopt formulation was fixed to a double-sided carbon tape. Then, it was kept on an aluminum stub and dried by vacuum. After that, LZIVSopt formulation was coated with gold using an ion sputter for 10 min up to 200 nm in an inert condition and captured the image [34] 

#### 2.4.4. Fourier-Transform Infrared Spectroscopy (FTIR)

FTIR spectra of the pure LZ and LZIVSopt formulation were recorded by FTIR (8400S, Shimadzu, Tokyo, Japan). Each sample was mixed in 1:100 with potassium bromide and compressed into pellets. The samples were scanned from 4000 to 400 cm^−1^ at room temperature [32]. 

#### 2.4.5. Differential Scanning Calorimetry (DSC)

DSC thermogram was performed using an instrument (DSC-60, Shimadzu, Tokyo, Japan). The 4 mg of the samples (pure LZ and LZIVSopt) was placed in an aluminum pan and examined at a scanning rate of 10 °C/min at a temperature range of 20–400 °C in an inert atmosphere maintained with nitrogen [35].

#### 2.4.6. Formulation of Luliconazole-Loaded Invasomes Gel

The selected optimized IVS formulation was converted into gel to improve transdermal delivery. The gel base was prepared by dispersing different concentrations of Carbopol 934 (1–2.5% *w*/*v*) in distilled water (Table 2). Propylene glycol and glycerin are added for hydration. Then the mixture was incubated overnight for complete swelling of Carbopol. The LZIVSopt formulation (1% LZ) was added to the gel base and neutralized with triethanolamine [36]. The transparent and clear gel was formed and stored at a cool condition for further analysis. 

### 2.5. Evaluation of Invasomes Gel

#### 2.5.1. Viscosity Determination

The viscosity of the prepared different LZIVSopt gels (G1–G4) was analyzed with a viscometer (Hakke G, D8, Mumbai, India). LZIVSopt gel was placed in a beaker, and its viscosity was measured by dipping the spindle (10S) at 50 rpm for 1 min at 25 °C.

#### 2.5.2. Evaluation of pH

The pH of the different LZIVSopt gels was evaluated by using a digital pH meter (LabIndia, Thane, Mumbai, India). The 1 g of each LZIVSopt gel was dispersed into 100 mL of double distilled water. The dispersion was stood for 1 h for equilibrium to be attained, and the pH was recorded by dipping the electrode for 1 min at room temperature [37].

#### 2.5.3. Drug Content

The drug content of LZ in LZIVSopt gels was determined by dissolving 1 g of prepared LZIVSopt gel in methanol. The sample was filtered through a membrane filter (0.45 µm). Then, the absorbance was measured using UV- spectrophotometry at 297 nm after appropriate dilution with phosphate buffer (pH 7.4).

#### 2.5.4. Spreadability

The spreadability of the LZIVSopt gels was measured by the slip and drag method [38]. The LZIVSopt formulation was placed between two glass slides of the same dimension. Then, a constant weight (200 g) was applied to the upper glass slide for 5 min to remove the air from the gel. Then, the upper slide was bound with thread and supported with wood pully. Then, 40 g of weight was applied with the thread of pully, and the upper slide was moved over the gel for a certain distance. the time required for the gel to move the distance of the place from its original position was noted. The spreadability of the gel was calculated by the following equation.

(2) $$Spreadability=\frac{Weight\;place\;on\;pully\times lenght\;of\;slide\;}{Time\;(Sec)}$$

#### 2.5.5. Animals Handling

The study was conducted on male adult rats (150–200 g, Albino Wistar rats). The study protocol was approved by Jeeva life sciences Hyderabad, India (CPCSEA/IAEC/JLS/17/03/22/047). The study was conducted as per ARRIVE guidelines. Rats were in-house at 22 ± 1 °C and 12/12 h dark/light cycle for 2 weeks and free-fed a normal diet and tap water.

#### 2.5.6. Evaluation of Ex Vivo Drug Release of Invasomal Gel

Ex vivo permeation study of the optimized LZIVSopt gel was performed using excised rat skin and compared with pure LZ-gel and LZIVSopt. The study was performed using Franz diffusion cells under simulated skin pH conditions. The rats were kept in fasting condition overnight before the study. The rats were sacrificed by using a diethyl ether inhaler and cervical dislocation, and the abdominal skin was collected [39]. The hair was removed with hair removal cream carefully without any damage and cleaned with phosphate buffer saline (PBS). The skin was placed between the donor and acceptor compartments of the diffusion cell, and the stratum corneum faced over the donor compartment. The 15 mL of the PBS solution (pH 7.5) was filled into the acceptor compartment, and the touch of the skin layer was assured and maintained the 37 ± 0.5 °C during the whole period. The medium was rotated at 50 rpm. The required quantity of LZIVSopt, LZIVSopt gel, and pure LZ-gel (3 mg of LZ) was filled into the donor compartment. An volume of 1 mL of the sample was withdrawn at a fixed time (0, 1, 2, 3, 4, 6, 8, 12, and 24 h), and the fresh medium was added into the acceptor compartment simultaneously to maintain the constant volume. The sample was filtered through the membrane filter (0.45 µm), and the concentration was analyzed through the previously reported validated HPLC method [37]. The mobile phases consist of methanol and acetonitrile (45:55 *v*/*v*) and flow at a flow rate of 0.75 mL/min. Detection was performed using a UV detector at 297 nm, and the run time was 10 min. The % permeation, flux, and apparent permeability coefficient were calculated.

(3) $$APC=\frac{Flux\;}{Initial\;concentration\times \;diffusion\;area}$$

#### 2.5.7. Examination of Skin Irritation

The skin irritation study was conducted using Wistar albino rats. The study was carried out in accordance with regulations and institutional animal ethics committee approval (IAEC). The hair of the dorsal side skin, which is about 4 cm^2^ area of the skin, was removed using hair trimmer and a sterilized razor. The rats were randomly divided into three groups: group 1 for the control, group 2 for the LZIVSopt gel, and group 3 for the negative control (0.9% NaCl). The formulations were applied over the skin of the respected group and covered with patches. After that the skin was washed with PBS and at definite time intervals, the responses were recorded by visual observation for a sign of erythema, edema, and any damage to the skin up to 24 h [40].

#### 2.5.8. In Vivo Antifungal Activity

The in vivo anti-fungal activity was conducted in rats by infecting them with Candida albicans (*C. albicans*). The Sabouraud’s dextrose agar medium was prepared and inoculated with *C. albicans* for growth. The plate was kept in an incubator at 30 °C for 48 h. The *C. albicans* colony was collected and suspended in sterile phosphate buffer saline (PBS), and the final concentration is 10^7^ CFU/mL. The hair was removed on the dorsal side skin of the rats (2 cm^2^) by using a hair trimmer and cleaned with PBS. The rats were divided into three groups: group 1 for infected control, group 2 for pure LZ-gel, and group 3 for LZIVSopt gel (25 mg/kg/day LZ). The 100 µL of *C. albicans* suspension was gently applied through a cotton-tipped swab onto the skin of rats of the respected groups (groups 1–3). The infection was checked after 48 h inoculation. Then, the treatment was started with 25 mg/kg of LZ one dose a day for 14 days in the respective group (group 2 and group 3). The infected rats were sacrificed at the end of the protocol (last dose) for *C. albicans* evaluation. The infected area of the skin was excised and washed with sterile PBS, then inoculated into Sabouraud’s dextrose agar plates under aseptic conditions and stood for 1 h at 25 °C, and then incubated for 48 h at 37 °C and recorded the CFU value [41]. 

### 2.6. Statistical Evaluation

The statistical design was used for the optimization of IVS. GraphPad prims-9 (San Diego, CA, USA) was used for statistical comparison analysis (one-way ANOVA, student *t*-test). *p* < 0.05 was taken as for consideration of the significant difference.

## 3. Results and Discussion

### 3.1. Optimization of Nano Invasomes

**Figure 1:**
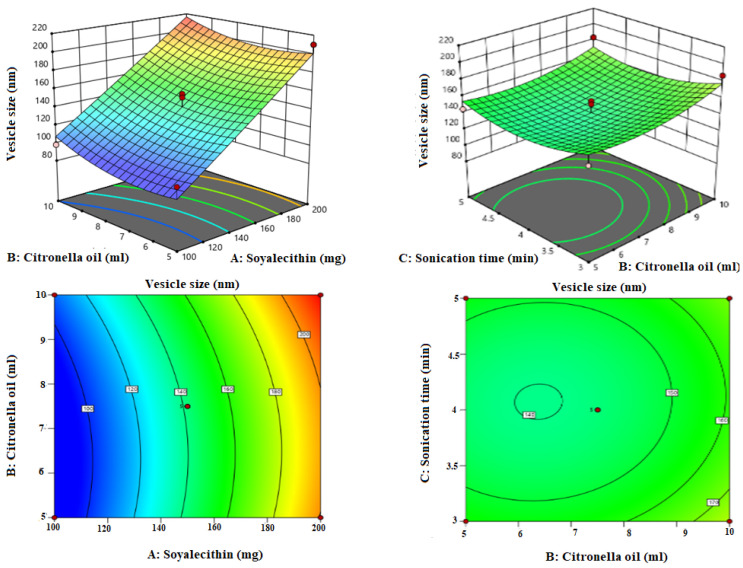
Response surface plots explaining the effect of citronella oil, soy lecithin, and sonication time over the vesicle size of invasomes. Citronella oil and soy lecithin showed positive effects, and sonication time showed negative effects on the vesicle size.

**Figure 2:**
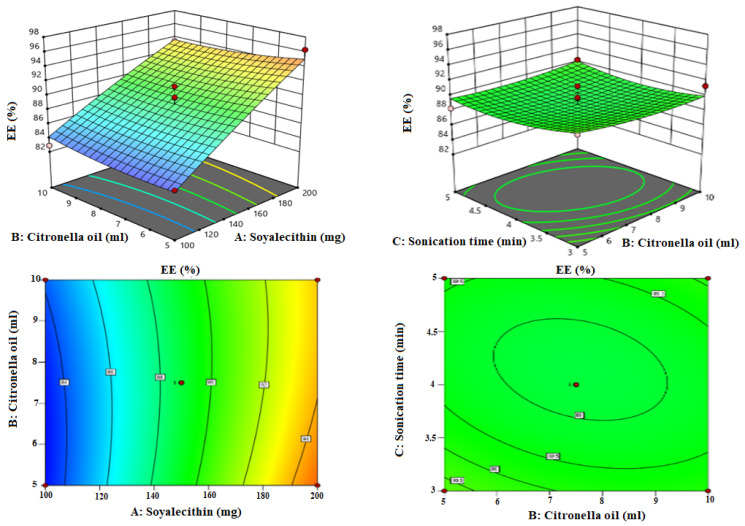
Response surface plots explaining the effect of citronella oil, soy lecithin, and sonication time over the entrapment efficiency of LZ in invasomes. Citronella oil and soy lecithin showed positive effects, and sonication time showed negative effects on the entrapment efficiency of LZ in invasomes.

**Figure 3:**
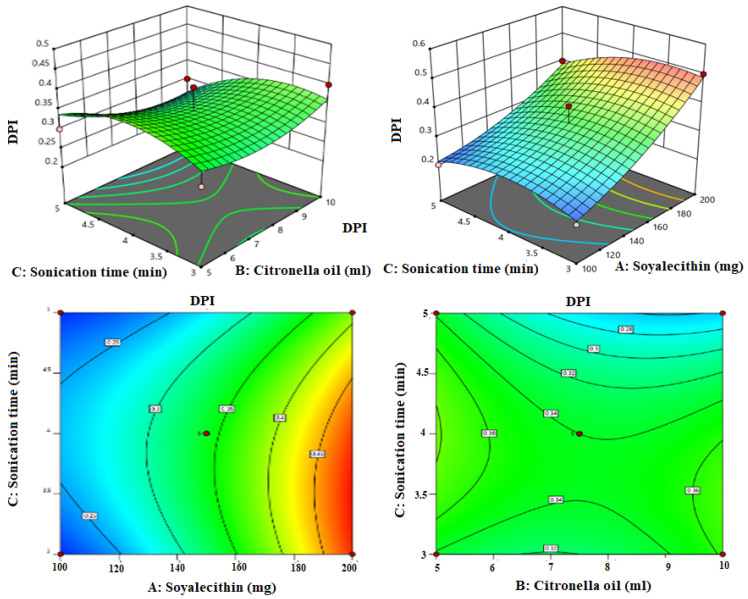
Response surface plots explaining the effect of citronella oil, soy lecithin, and sonication time over the PDI of invasomes. Citronella oil and soy lecithin showed positive effects, and sonication time showed negative effects on the PDI of vesicle size.

Box-Bhekhen design was used for the optimization of LZIVS. The composition and result of the response of thirteen formulations are shown in Table 1. The values of responses (VS, EE, and DPI) were fitted in linear, 2nd order, and quadratic models. The quadratic model was found to be the best fit for all responses because of the maximum R^2^ (0.9654 for VS, 0.9732 for EE, and 0.9603 for PDI) compared with other models. The response surface plot (3D and contour plot) was constructed (Figure 1, Figure 2 and Figure 3) which explained the effect of independent variables (Phospholipid, Terpene oil, and Sonication time) over the responses (VS, EE, and PDI).

### 3.2. Influence of Independent Factor on Vesicle Size (Y_1_)

VS of IVS is a crucial factor because it directly influences the penetration as well as absorption of therapeutic agents. The VS 600 nm or >600 nm are not easily penetrated deeper layers of the skin. The polynomial equation of the best-fitted model was constructed and is given below.

Y_1_ = +141.80 + 55.75A + 9.13B − 2.38C − 1.25AB − 0.25AC − 1.00BC + 0.85A^2^ + 10.10B^2^ + 13.10C^2^

The A, B, and C are the model terms of independent variables. The positive and negative signs of the polynomial equation denote the favored and unfavored effect of independent variables over the dependent variables, respectively. The quadratic model is significantly best fitted (*p* < 0.0001, R^2^ = 0.9654) for VS, their lack of fit is non-significant (*p* = 0.490), and it is good for the model. The 3D and contour plots were constructed, which explained the effect of soy lecithin (A), Citronella oil (B), and sonication time (C) on the VS (Figure 1). The VS of all LZIVS formulations was in the range of 95.0 nm (LZIVS1)–217.0 nm (LZIVS3) (Table 1). Phospholipid (soy lecithin, A) exhibited a prominent positive effect on the VS of the IVS. The concentration of soy lecithin (A) increases the VS of the IVS increases because soy lecithin contains a single hydrophobic chain with a polar head group that mixes impulsively into the bilayer membrane, subsequently forming of extremely positive bend in the membrane and increasing the VS [42]. In addition, with the increase in the concentration of soy lecithin, the viscosity of the mixture increases, thereby increasing the VS [43,44]. Terpene oil (Citronella oil, B) concentration increases the VS, but it has less effect than soy lecithin. It may be due to the large amount of LZ encapsulated in IVS with terpenes, which leads to an increase in the size of the IVS [45,46]. On increasing the sonication time, the VS of LZIVS decreased due to of breaking of the vesicle.

### 3.3. Influence of Independent Factor on Entrapment Efficiency (Y_2_)

The polynomial equation of the quadratic model for EE is given as

Y_2_ = +88.84 + 5.35A + 0.07B − 0.25C − 0.62AB − 0.32BC + 0.32AC − 0.3A^2^ + 0.02B^2^ + 0.8425C^2^

The positive and negative signs denoted the favored and unfavored effect of the independent variables over the EE. The quadratic model is the best fit and significantly fitted (*p* < 0.05, R^2^ = 0.9732). The lack of fit is non-significantly fitted (*p* = 0.59, *p* > 0.05), which is good for the fitted model and indicates less dissimilarity among the actual and predicted value of the EE. The adequate precession is <4 (67.43), revealing close agreement between the actual and predicted value of the response as well as also establishing the validity of the model. The 3D and contour plots showed the effect of soy lecithin (A), Citronella oil (B), and sonication time (C) on the EE (Figure 2). The EE of LZ in LZIVS formulations was found in the range of 82.9% (LZIVS9) to 96.3% (LZIVS13) (Table 1). It was observed that soy lecithin shows a significant positive effect on the EE of LZ in IVS. However, the remaining variables (Citronella oil and sonication time) exhibited insignificant effects on EE of LZ in IVS. Increasing the concentration of soy lecithin increased the EE of LZ in LZIVS. It is due to increases in the solubility of LZ in the lipid matrix. In addition, increasing soy-lecithin concentration increases the LZ to integrate into lipid content and increases the solubility, hence increasing the EE [47]. These findings agree with previously reported research, i.e., Olmesartan medoxomil -loaded IVS [25] and methotrexate- IVS [33]. Citronella oil (B) showed a positive effect on EE. As the Citronella oil concentration increased, the EE of LZ in IVS increased because more chains are available, which improved the solubilization of the LZ [20]. A similar type of finding was reported in Dapsone-IVS [36]. The sonication time increases as the EE of LZ in LZIVS decreased due to the leaching drug from the vesicle.

### 3.4. Influence of Independent Factor on Polydispersity Index (PDI)

The polydispersity index is a ratio that provides details of the size distribution of particles (uniformity of particle) in a particular system. The polynomial equation of the fitted model for the polydispersity index is given below.

Y_3_(PDI) = +0.34 + 0.11A + 0.013B − 0.03C + 0.050AB − 0.025AC − 0.025BC + 0.032A^2^ + 0.030B^2^ − 0.04C^2^

The positive and negative signs of the polynomial equation represented the favor and un-favor of the independent variable (soy lecithin, Citronella oil, and sonication time) over the PDI of the vesicle. The quadratic model was found to be best fitted (*p* < 0.05, R^2^ = 0.9603). The Lack of Fit is non-significant (F-value 1.39, *p* > 0.05) suggesting that the model was successfully fitted and confirming the validity of the model. The adequate precession value is >4 (78) indicating that the fitted model has an adequate signal. The 3D and contour plots were constructed, which explained the effect of soy lecithin (A), Citronella oil (B), and sonication time (C) on the PDI (Figure 3). The PDI of all LZIVS formulations was found in the range of 0.262 (LZIVS1) to 0.482 (LZIVS13) (Table 1). The soy lecithin (A) exhibited a more prominent effect than Citronella oil (B) and sonication time (C). When increasing the concentration of soy-lecithin (A) and Citronella oil (B), the PDI of vesicles increases (Figure 3) due to the increased size of LZIVS. However, when increasing the sonication time, the PDI is decreased due to decreased the VS but less prominent effect than lipid.

### 3.5. Point Prediction Optimization

The optimized formulation was selected from the point prediction of the software after further modification in the composition of the center point formulation (LZIVS8). After further changes, the optimized formulation (LZIVSopt) has 148 mg of soy lecithin, 9.35 mL of Citronella oil, and 3.56 min of sonication time. The predicted value of the responses is 146.83 nm of VS, 85.99% of EE, and PDI of 0.322, respectively. The experimental value of the responses was found to be 139.1 ± 4.32 nm of VS, 88.21 ± 0.82% of EE, and 0.301 ± 0.032 of PDI. The result shows a small deviation between the experimental and predicted value of the responses. The desirability function value of the optimized formulation (LZIVSopt) was found to be 0.99, which confirmed the validity and robustness of the model. 

### 3.6. Vesicle Size, PDI, and Zeta Potential

**Figure 4:**
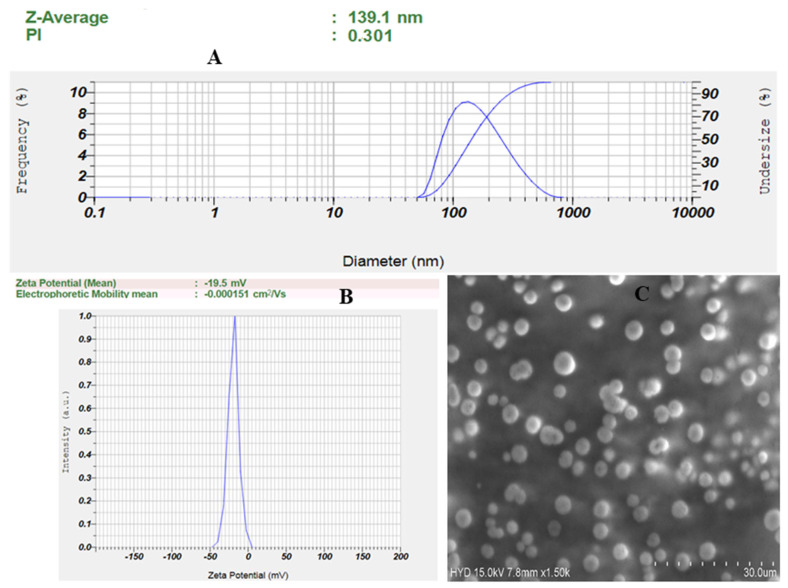
(**A**) Vesicle size and its distribution, (**B**) zeta potential graph, and (**C**) SEM image of the optimized invasomes (LZIVSopt).

The VS and PDI of the LZIVSopt are 139.1 ± 4.32 nm (Figure 4A) and 0.301 ± 0.032, respectively. The PDI is <0.5 indicating the homogeneous and uniform distribution of IVS. The zeta potential of LZIVSopt is 19.5 mV (negative) (Figure 4B) indicating high stability and disaggregated form of IVS.

### 3.7. Scanning Electron Microscopy

The shape of the LZIVSopt was analyzed with SEM, and the image is shown in Figure 4C. The vesicle was found to be spherical in shape and separated from each other. The image showed that vesicles were smooth, spherical, and free from the drug crystals.

### 3.8. FTIR Analysis

**Figure 5:**
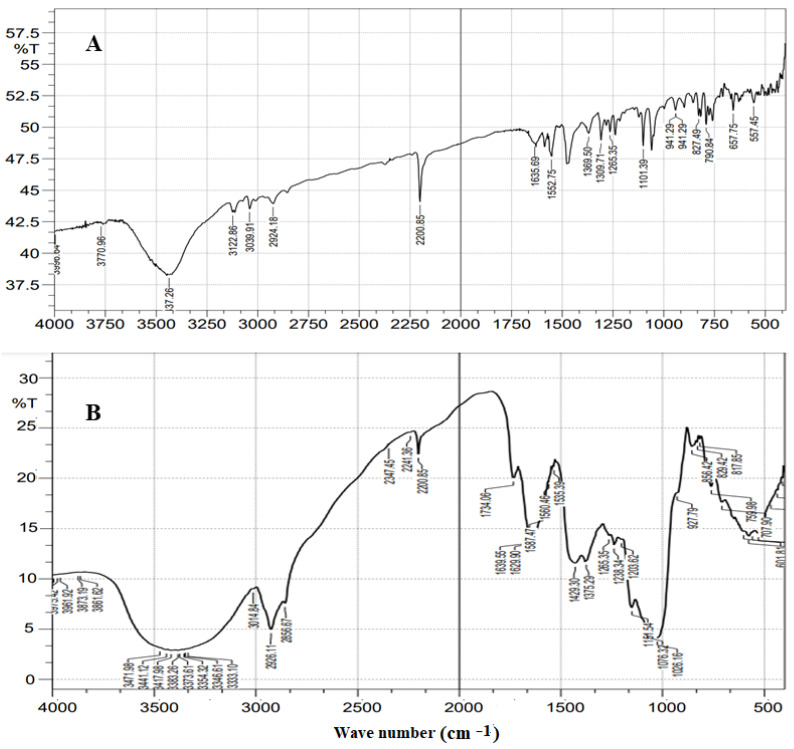
FTIR spectra of (**A**) pure LZ and (**B**) optimized formulation (LZIVSopt).

Figure 5 shows the FTIR result of pure LZ and LZIVSopt formulation. The spectra of LZ showed the characteristic absorption at 3437.26 cm^−1^ (C–H stretching, aromatic), 2924.18 cm^−1^ (C-H aliphatic stretching), 2200.85 cm^−1^ (C≡N stretching), 1635.69 cm^−1^ (C=C alkene stretching), and 1552.75 cm^−1^ (C=C stretching aromatic) respectively, indicating the purity of the drug (Figure 5A). The spectra of LZIVSopt showed the peaks at 3471 cm^−1^ (O–H stretching vibration), 1734.06 cm^−1^ (C=O stretching vibration), 1151.54 cm^−1^ (C–O stretching vibration), and 1076.32 cm^−1^ (C–C stretching vibration), representing the characteristic peaks of phospholipid (soy-lecithin). The same absorption peaks of LZ were found in the FTIR spectra of LZIVSopt (Figure 5B). It revealed that there is no chemical interaction between LZ and excipients and they are compatible with each other. Similar types of observation were reported in Glibenclamide and Atenolol combination IVS [32] and in the tizanidine-loaded IVS for topical delivery [48]. 

### 3.9. Differential Scanning Calorimetry 

**Figure 6:**
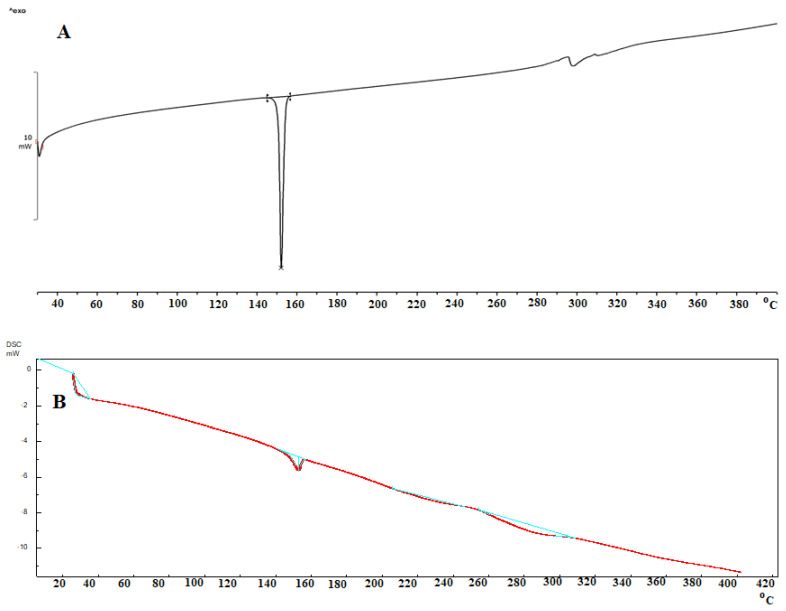
DSC spectra of (**A**) pure LZ showing a sharp endothermic peak at melting point (150.40 °C) and, (**B**) optimized formulation (LZIVSopt) did not show a peak of LZ (different colors explain the peaks).

The DSC of pure LZ and LZIVSopt was performed for analysis of crystallinity, and the results are shown in Figure 6. The thermogram of LZ showed an intense peak at 150.40 °C, revealing its crystalline nature and purity of LZ (Figure 6A). However, the thermogram of the LZIVSopt did not show any characteristic peaks of LZ (Figure 6B), confirming that the LZ was encapsulated into the lipid matrix of IVS. A similar type of observation was reported in olmesartan medoxomil and Dapsone-loaded vesicle preparation for transdermal delivery [35,49]. Another study reported that IVS loaded with propranolol hydrochloride did not show any sharp peaks, revealing that the drug was encapsulated inside of INV or transformed into an amorphous form [50].

### 3.10. Development of Luliconazole-Loaded Invasomes Gel

The LZIVSopt was successfully incorporated into the different concentrations of Carbopol 934 gel and evaluated for different parameters. 

### 3.11. Viscosity, pH, and Drug Content

Table 3 shows the results of the evaluation parameters of all LZIVSopt gel formulations. The consistency of the gel is very important because it is applied on the skin in a thin-layer form. The viscosity of the gel has played an important role in controlling drug permeation in the skin [51]. The viscosity of all formulations was found in the range of 5358 ± 43 cps (LZIVSopt-G1) to 6671 ± 37 cps (LZIVSopt-G4). The viscosity of the gel increases with the increase in the polymer concentration due to increased gelling strength. The pH of all LZIVSopt gel was found within the skin pH range, indicating compatibility with skin, and it can be used for ex vivo and in vivo evaluation. The drug content of all LZIVSopt gels was found to be 98.86–99.60%, and there is no significant difference between each batch of the formulation.

### 3.12. Spreadability

The spreadability assists in the consistent application of gel to the skin and greatly impacts patient compliance and therapeutic efficacy. An optimum value of spreadability indicates the gel spreads appropriately in less time and stays for a longer time over the skin [46]. The spreadability of all LZIVSopt gel was determined, and the result is shown in Table 3. The spreadability value of all LZIVSopt gels is in the range of 6.1 to 8.5 g·cm/s. The spreadability of gel decreases by increasing the polymer concentration due to an increase in viscosity. This result agreed with the previously reported finding of topical itraconazole-encapsulated lipid gel [18].

### 3.13. Selection of Optimized LZIVSopt Gel

On the basis of the optimum viscosity and spreadability of the gel, the LZIVSopt-G3 formulation was selected as an optimized gel and used for further study. The LZIVSopt-G3 has a viscosity of 6493 ± 27 cps and 7.2 g·cm/s of spreadability.

### 3.14. Ex Vivo Diffusion Study

**Figure 7:**
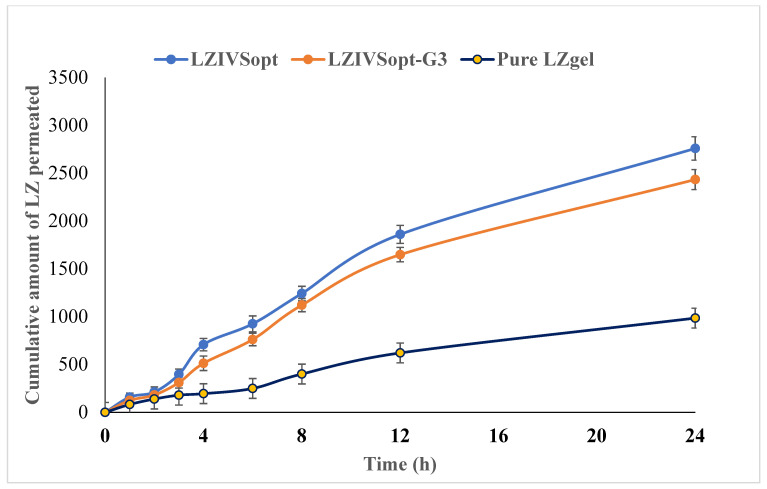
Ex vivo permeation study graph of the different formulations using excised rat skin through Franz diffusion cell.

Ex vivo diffusion study was performed using the excised rat skin, and the % LZ permeated from pure LZ-gel, LZIVSopt dispersion, and LZIVSopt-G3 are shown in Figure 7. The % LZ permeation from pure LZ-gel, LZIVSopt dispersion, and LZIVSopt-G3 are 984.83 ± 95.00 µg (32.83 ± 3.17%), 2758.06 ± 123.00 (91.94 ± 4.10%), and 2433.10 ± 105.00 µg (81.10 ± 3.50%), respectively, in 24 h. The flux of all formulations was calculated and found to be 41.0 µg/cm^2^·h for pure LZ-gel, 118.96 µg/cm^2^·h for LZIVSopt, and 106.42 µg/cm^2^·h for LZIVSopt-G3, respectively. The APC of pure LZ-gel, LZIVSopt dispersion, and LZIVSopt-G3 were found to be 3.62 × 10^−4^ µg/min, 9.38 × 10^−4^ µg/min, and 1.04 × 10^−3^ µg/min, respectively. LZIVSopt and LZIVSopt-G3 exhibited significantly (*p* < 0.05) more permeation of LZ, i.e., 2.90-fold and 2.60-fold of LZ-gel. The ER ratio of LZIVSopt-G3 was 2.60 as compared to pure LZgel, demonstrating significantly high permeation through the skin. The high permeation of LZ through the rat skin from LZIVSopt and LZIVSopt-G3 is credited to the coactive effect of citronella oil and alcohol. The alcohol provided the fluidity of the lipid layer of the vesicle, and the soft structure leads to enhanced permeation. However, citronella oil fluidized the stratum corneum lipid layer and disturbed lipid arrangement, thus smoothing the permeation of IVS [45]. In addition, higher permeation is also due to the nanosize of IVS [20]. Nemr’ group formulated Cilostazole-loaded vesicular gel for topical delivery and showed a significant enhancement ratio to pure drug dispersion [52].

### 3.15. Skin Irritation Study

**Figure 8:**
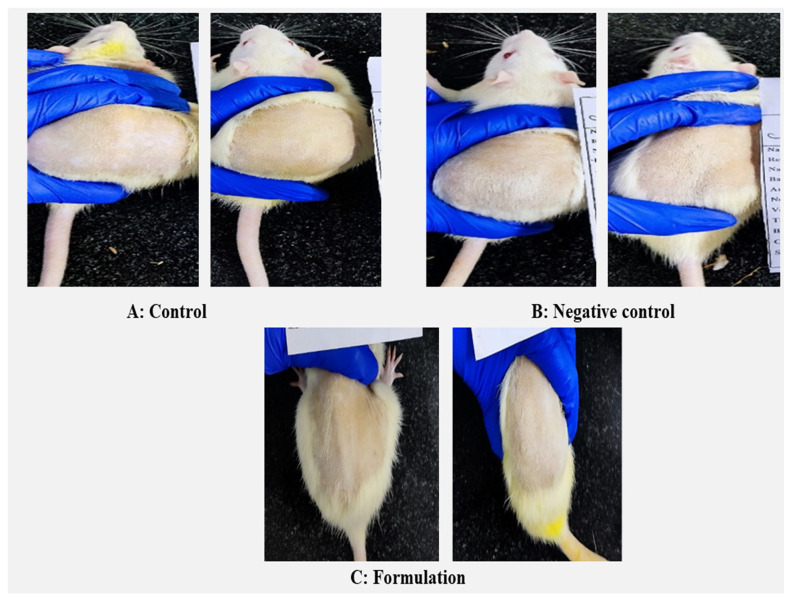
The irritation study of (**A**) control: without treatment, (**B**) treated with 0.9% sodium chloride, and (**C**) treated with optimized formulation (LZIVSopt) in albino Wistar rats.

The skin irritation of the LZIVSopt-G3 was carried out on rat skin and compared with normal control and negative control (0.9% NaCl) groups, and the result is depicted in Figure 8. There is no irritation (erythema and edema, score 0) was found in the rats after the treatment with LZIVSopt-G3 for 24 h of the study and similar to normal control and negative control (0.9% NaCl) (Figure 8A–C). It revealed that IVS gel was stable and not producing any toxic effect on the rat skin.

### 3.16. In Vivo Antifungal Activity

**Figure 9:**
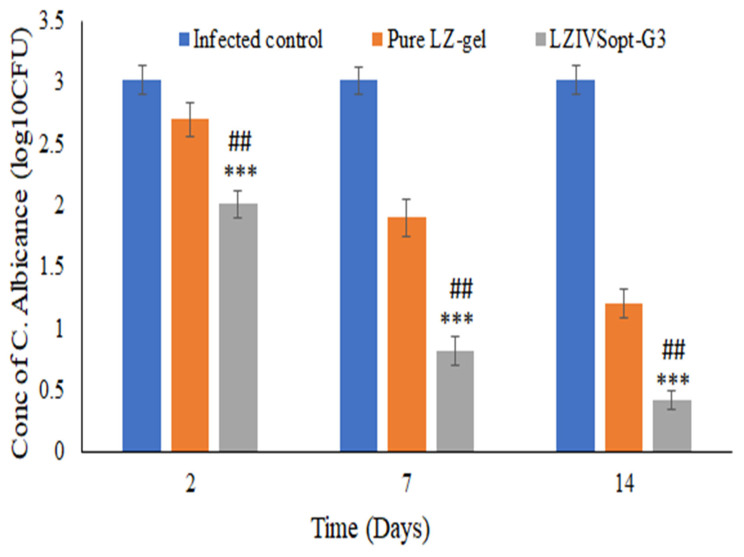
In vitro antifungal activity against *C. albicans* of LZIVSopt-G3 and compared with pure LZ-gel and infected control. The figure showed the concentration of C. Albicans at different time intervals. ## (*p* < 0.01) compared with pure LZ-gel, *** (*p* < 0.001) compared with infected control.

The in vivo antifungal activity of LZIVSopt-G3 and pure LZ gel was carried out in albino Wistar rats after infecting the fungal stain (*C. albicans*) and compared to the infected control group (no treatment). The result is shown in Figure 9 at different time intervals. All the infected rats showed 3.02 ± 0.12 log_10_ CFU of *C. albicans* before the treatment. LZIVSopt-G3 and pure LZ-gel exhibited a significant decrease (*p* < 0.05) in the *C. albicans* (CFU) up to 14 days of treatment as compared to the infected control. However, LZIVSopt-G3 exhibited a significantly (*p* < 0.05) high antifungal effect than pure LZ-gel. The high activity of LZ by the IVS-gel is due to increases in solubility, high penetration, and high accumulation of IVS in rat skin. This effect is due to the presence of alcohol and citronella oil and nanosized vesicles. The high activity is also due to sustained releases of LZ from the LZIVSopt-G3 [53].

## 4. Conclusions

The present research work was to develop LZIVS by thin-film hydration method and optimized by using the Box-Bhekhen design software. LZIVS showed the nano-size range of vesicles (139.1 ± 4.32 nm) with high homogeneity (<0.301 ± 0.032 of PDI). The zeta potential was found to be highly negative (19.5 mV) revealing high stability and non-aggregated vesicles. The LZIVSopt showed high entrapment of LZ (88.21 ± 0.82%). DSC study of LZIVSopt did not show LZ peak revealing it encapsulation into invasomes matrix. Further, LZIVSopt was successfully converted into carbopol gel. LZIVSopt gel showed good consistency and excellent spreadability. LZIVSopt gel exhibited significantly (*p* < 0.05) high permeation (81.10 ± 3.50%) through excised rat skin. LZIVSopt gel did not show any irritation (swelling, erythema) in the rat skin during the 24 h of the study. LZIVSopt gel showed a significantly (*p* < 0.05) higher in vivo antifungal effect than LZ-gel. The finding concluded that IVS gel is a good carrier for LZ for transdermal delivery to treat the topical fungal infection.

## Figures and Tables

**Table 1:** Composition of various invasomes formulations obtained from Box-Bhekhen design software and data of responses (vesicle size, entrapment efficiency, and PDI).

Formulation Trail	Phospholipid, (A, mg)	Terpene Oil (B, ml)	Sonication Time (C min)	Vesicle Size, (nm, Y_1_)	Entrapment Efficiency (%, Y_2_)	PDI (Y_3_)
LZIVS1	100	7.5	5	95	85.2	0.262
LZIVS2	100	7.5	3	102	83.9	0.273
LZIVS3	200	7.5	3	217	94.2	0.473
LZIVS4	150	10	3	187	91.3	0.432
LZIVS5	150	5	5	145	88.3	0.321
LZIVS6	200	10	4	204	93.4	0.452
LZIVS7	200	7.5	5	209	94.2	0.412
LZIVS8	150	7.5	4	156	89.8	0.301
LZIVS9	100	10	4	98	82.9	0.272
LZIVS10	150	5	3	163	90.6	0.342
LZIVS11	100	5	4	107	83.3	0.274
LZIVS12	150	10	5	183	90.3	0.383
LZIVS13	200	5	4	210	96.3	0.482

**Table 2:** The composition of different luliconazole invasomes-laden gels.

Composition	LZIVSopt-G1	LZIVSopt-G2	LZIVSopt-G3	LZIVSopt-G4
Carbopol 934	1	1.5	2	2.5
Triethanolamine	0.5%	0.5%	0.5%	0.5%
Distilled water (QS)	100 mL	100 mL	100 mL	100 mL

**Table 3:** Results of physicochemical characterization parameters of different developed luliconazole-incorporated invasomes gel.

Formulation Code	pH	Viscosity (cps)	Drug Content (%)	Spreadability (g·cm/s)
LZIVSopt-G1	6.4 ± 0.3	5358 ± 43	98.86 ± 1.32	8.5
LZIVSopt-G2	6.4 ± 0.2	6028 ± 25	98.92 ± 1.13	7.8
LZIVSopt-G3	6.5 ± 0.3	6493 ± 27	99.60 ± 1.02	7.2
LZIVSopt-G4	6.2 ± 0.4	6671 ± 37	99.31 ± 1.52	6.1

## Data Availability

The data can be provided on request from the corresponding author.
